# A Double Tragedy

**Published:** 1888-09

**Authors:** 


A DOUBLE TRAGEDY.—In a fit of temporary insanity Dr. Archibald G. Paddock, a retired New York dentist, shot four bullets into the body of his son, and then blew off the top of his own head. Both men died almost instantly. The doctor was well known in New York, having practiced there for many years previous to his coming here. His family consisted of himself, his wife, a young daughter and a boy, Harry 18 years of age.

The father fairly worshiped the son and took him with him everywhere. The boy had recently decided to leave home and had secured a position with a firm in New York, where he was to begin work Monday morning. Dr. Paddock and Harry were both experts in the use of firearms and spent much of their time in rifle and pistol practice. They went out about 9 o'clock in the morning and rigged up a target in the rear of the barn. They had been shooting for an hour, when the doctor was called to the house to pull a tooth. He returned to the target and ten minutes later both he and his son were stretched dead on the field.

There was but one eye witness to the murder, Samuel S. Dauchy, and his rear lot joins that of the doctor. This is the story he tells:

"I was out in my garden picking beans when the shooting was done. I saw the doctor and his son come out, the doctor carrying the guns. I did not notice them particularly because I've seen them shooting at a target several times before. The first I saw peculiar was that the doctor loaded and fired into the ground. He did that, I should think, half a dozen times, then he fired once into the air. It was quite early in the day when that took place, and afterwards I saw the doctor had gone away."

"About 11 I saw Harry moving away from his father. The doctor had his right arm extended, and I presume his pistol was in his hand. I was so startled I did not notice the pistol was pointed right at the boy. There had been one report, and just as I looked the pistol was discharged again and the boy fell. He was only about ten feet away from his father. It was terrible, but what followed was even more frightful. The old man quietly picked up the shot gun, placed the stock on the ground, and laid the left side of his face across the muzzle. How he pulled the trigger I don't know, but there was a loud report, and the old man staggered and fell backward."

When found both men were lying on the ground about eight feet apart. Both were dead. Dr. William S. Todd, county medical examiner, made an examination of the bodies. There were four bullet holes in the boy's body, all from the pistol. One had struck the face just below the left eye, and had entered the brain diagonally. The other struck further up and a little to the side of the head. The body wounds were in the vicinity of the heart, and judging from their general direction, had been made while the boy was standing with his left side towards his father.

Dr. Paddock was born in Putnam county, N. Y., but had been known in Ridgefield for more than twenty years. He married a Miss Sproull, of this place, and has lived here a greater part of the time since. For ten years he was with the Colton Dental association in Cooper Union, New York. He is a brother-in-law of Dr. J. C. Sproull, the dentist, of No. 667 Madison avenue, New York. Another brother-in-law is W. O. Seymour, a surveyor, of this town. The latter says of the tragedy:

"The doctor was of an exceedingly nervous temperament and of late he had not been very strong. He had never, however, shown the slightest symptoms of insanity. He was in comfortable circumstances and had nothing to worry him. That boy Harry was his idol. For many years he had been always at his elbow, and the two consulted together on every little topic that entered into their daily life. The doctor felt awfully homesick to think of his boy going out into the world and away from him. In my opinion this overbalanced his reason and caused hin to fire the fatal shots."

